# Behavior change techniques in a care partner–assisted oral health intervention for people with mild dementia: a secondary mixed-methods analysis

**DOI:** 10.1093/geroni/igag069

**Published:** 2026-06-25

**Authors:** Jing Wang, Courtney Caiola, Chiao-Hsin Teng, Youngmin Cho, Zhijing Xu, Jackie Finik, Shahrzad Siamdoust, Ya-Ning Chan, Eunjung Ko, Justine Seidenfeld, Ayomide Okanlawon Bankole, Donald E Bailey, Cappye Mott, Bei Wu, Brenda L Plassman, Ruth A Anderson

**Affiliations:** College of Health and Human Services, University of New Hampshire, Durham, New Hampshire, United States; College of Nursing, East Carolina University, Greenville, North Carolina, United States; School of Nursing, College of Medicine, Chang Gung University, Taoyuan, Taiwan; School of Nursing, Johns Hopkins University, Baltimore, Maryland, United States; School of Nursing, Montclair State University, Montclair, New Jersey, United States; Department of Family Science and Human Development, College for Community Health, Montclair State University, Montclair, New Jersey, United States; Rory Meyers College of Nursing, New York University, New York, New York, United States; Rory Meyers College of Nursing, New York University, New York, New York, United States; Department of Population Health Sciences, Duke School of Medicine, Durham, North Carolina, United States; Rory Meyers College of Nursing, New York University, New York, New York, United States; Department of Emergency Medicine, Duke University, Durham, North Carolina, United States; School of Nursing, Duke University, Durham, North Carolina, United States; School of Nursing, Duke University, Durham, North Carolina, United States; Department of Psychiatry, Duke University, Durham, North Carolina, United States; Rory Meyers College of Nursing, New York University, New York, New York, United States; Faculty of Arts and Science, NYU Shanghai, Shanghai, China; Department of Psychiatry, Duke University, Durham, North Carolina, United States; School of Nursing, Duke University, Durham, North Carolina, United States

**Keywords:** Oral hygiene, Dyadic intervention, Caregiving, Alzheimer’s disease

## Abstract

**Background and Objectives:**

Oral hygiene declines early in mild dementia (MD), yet few interventions specify which behavior change techniques (BCTs) drive improvement. We examined the use of BCTs in relation to oral health outcomes in a care-partner–assisted intervention for persons living with MD.

**Research Design and Methods:**

Using a secondary convergent mixed-methods design, we analyzed the coaching arm of a three-arm randomized trial. Seventeen dyads (persons living with MD-care partners) completed four coaching sessions (68 coaching session transcripts). Seventeen BCTs were specified for use in the coaching manual. Transcripts were double-coded to denote frequency of BCTs used. Changes in the Gingival Index (GI) of persons living with MD between pre- and post-intervention were categorized as *Stable/Worsened*, *Improved*, or *Meaningfully Improved*. BCT frequencies were compared across these categories and integrated with qualitative data using joint displays.

**Results:**

The most frequently used BCTs were reviewing behavior goals, problem-solving, behavioral instruction, and prompts/cues. Two unspecified BCTs (action planning and emotional/relational support) were also commonly used. Meaningfully Improved dyads showed coordinated routines, greater autonomy for persons living with MD, and flexible BCT adaptation; Improved dyads used iterative action planning; Stable/Worsened dyads relied more on coach-led BCTs with limited adaptation. Care partners most often delivered prompts/cues and problem-solving, whereas persons living with MD engaged in goal setting and skill acquisition.

**Discussion and Implications:**

While planned instructional BCTs established a necessary foundation, adaptive elements (e.g., action planning and relational support) proved essential for integrating oral care into daily life and achieving meaningful health improvement.

Innovation and Translational Significance: This study is among the first to systematically map specific behavior change techniques (BCTs) to clinical oral health outcomes in persons living with mild dementia using a mixed-methods design. By linking BCT frequency and dyadic role patterns to changes in Gingival Index, we identify not only core manualized techniques but also emergent adaptive strategies: action planning and relational support, as critical active ingredients. Findings advance translational dementia care by operationalizing BCTs as scalable, dyadic tools that can be embedded into daily routines and tested in larger trials to improve sustainable self-care.

Poor oral health is a prevalent and under-addressed issue among persons living with mild dementia (MD). Compared to cognitively intact older adults, those living with MD have more dental plaque, higher rates of caries, more severe periodontal disease, fewer teeth, and poorer oral health-related quality of life (Delwel et al., 2018; Lee et al., 2013, 2016; Maldonado et al., 2018). These outcomes are closely related to a decline in daily oral hygiene practices, particularly tooth brushing, which has been shown to mediate the relationship between cognitive impairment and oral health (Lee et al., 2016).

Oral hygiene decline in dementia is not simply a matter of motivation or health knowledge. Oral self-care depends on multiple neurocognitive processes, including memory, attention, sequencing, executive function, language comprehension, and praxis. Cognitive impairment can therefore disrupt not only whether oral hygiene occurs, but also how effectively it is performed (Chen et al., 2015). Despite these challenges, persons living with MD still typically retain the ability to do familiar and well-learned tasks, such as tooth brushing. Research indicates that even as other cognitive domains deteriorate, procedural memory tends to remain intact in the early stages of dementia (De Wit et al., 2021). It offers an important entry point for intervention. That is, with appropriate environmental support and guidance, persons living with MD can maintain oral hygiene behaviors to some extent, even if the quality of execution of the skills diminishes over time. To date, very few oral health interventions have been designed specifically for people living with dementia. A systematic review found that only two studies focused specifically on persons living with MD, and of the studies that included this population, all participants were living in residential care settings; none targeted those in the community (Siegel et al., 2017). Furthermore, these studies focused on oral health outcomes such as plaque levels rather than evaluating what and how oral hygiene behaviors changed or were sustained.

In home- and community-based settings, oral hygiene care among persons living with MD is rarely sustained through individual effort alone; rather, it is shaped within an ongoing caregiving relationship. Daily oral hygiene care often depends on spouses, adult children, or other informal care partners who provide reminders, cueing, supervision, and, at times, hands-on assistance (Anderson et al., 2019; Wu et al., 2022). This caregiving context is especially important in dementia, where cognitive changes can disrupt oral self-care by interfering with routine formation, sequencing of tasks, and motivation. Prior work shows that care partners play a central role in supporting oral hygiene, yet many report limited knowledge, training, and confidence in how to assist effectively with oral health care (Prieto et al., 2025). Thus, oral hygiene interventions for persons living with MD should be understood not simply as individual behavior-change programs, but as dyadic caregiving interventions embedded in the everyday realities of chronic illness management. From this perspective, oral hygiene challenges arise not only from the abilities of the person living with dementia, but also from how care partners interpret needs, provide support, negotiate resistance, and adapt strategies over time.

The literature suggests that to maximize the effectiveness of such interventions, it is essential to specify the behavior change techniques (BCTs) that serve as the intervention’s active components. BCTs are defined as systematic procedures specified as active components to facilitate behavior change in interventions (Michie et al., 2013). These BCTs, such as feedback, self-monitoring, and reinforcement, are observable, replicable, and irreducible (Michie et al., 2013). While BCTs have proven effective across a range of health contexts, including diet and diabetes management (Cradock et al., 2017; Samdal et al., 2017), their systematic application to oral hygiene care, and particularly to dementia contexts, remains limited and insufficiently described. As emphasized by Asimakopoulou et al. (2023), it is essential to demystify behavioral science and offer practical tools to deliver oral health behavior change. Specifically, investigating what BCTs work best for which oral health behaviors and in which settings, and identifying who is best placed to support behavior change and how, are critical research questions in the current state of behavioral science (Asimakopoulou et al., 2023).

Applying BCTs to persons living with MD, however, requires careful tailoring. Cognitive impairments inherent to dementia can limit the effectiveness of techniques (e.g., action planning or self-monitoring behaviors) that rely on complex cognitive processes (Nyman, 2019). Partnering with care partners offers a viable solution: care partners can facilitate BCTs, providing problem-solving, instructions, and feedback on behavior. A commentary on oral-health behavioral change argues that one-way information is insufficient; sustained behavioral change requires interactive BCTs—real-time demonstration, iterative feedback, and personalized support (Asimakopoulou et al., 2023). Because these techniques must be woven into daily routines, care partners are ideally positioned to provide real-time observation, immediate feedback, and ongoing motivation. However, little is known about how care partners implement BCTs and the extent to which BCTs influence health outcomes among persons living with MD.

A prior feasibility study aimed to improve oral hygiene and oral hygiene behaviors among persons living with MD (Wu et al., 2021) demonstrated the potential of incorporating BCTs into care partner-assisted interventions. Each dyad of persons living with MD and their care partners received a tailored care plan, an educational booklet, and four coaching sessions for care partners about how to assist those living with MD. In this pilot study, persons living with MD in the intervention group showed significant improvements (e.g., a greater reduction in plaque levels, gingival inflammation, and overall oral health status) compared with those in the control group (Wu et al., 2021). The qualitative analyses of the pilot study data showed that the most frequently used BCTs among care partners were prompts/cues, instructions on how to perform the behavior, reviewing behavioral goals, problem-solving, and using credible sources (Bryant et al., 2024). Building on these findings, this study aimed to examine how care partners’ use of BCTs influenced oral health outcomes among persons living with MD. Specifically, we compared changes in the oral health outcome (i.e., Gingival Index) across three outcome groups (Stable/Worsened, Improved, or Meaningfully Improved) and explored similarities and differences in BCT use across these groups.

## Method

### Study design

In the parent study, persons living with MD were randomized using a block randomization method developed in advance by the study’s statistician (Wu et al., 2021) to (1) control, (2) Treatment 1 (electric toothbrush only), or (3) Treatment Group 2 (electric toothbrush and a full care-partner–assisted intervention in which the care partner learned to facilitate the oral hygiene behaviors of persons living with MD). This study is a secondary convergent mixed-methods analysis of data drawn from the coaching arm of a three-arm randomized controlled trial. Using a convergent mixed-methods design (Fetters et al., 2013), this analysis focused specifically on BCTs used during intervention coaching sessions, and thus, we included only participants enrolled in Treatment Group 2. We used mixed methods because neither strand alone was sufficient to answer the study question: quantitative coding captured the frequency and distribution of BCTs, while qualitative analysis was needed to examine how these techniques were enacted, adapted, and embedded within dyadic caregiving interactions. The qualitative and quantitative strands were analyzed in parallel and integrated through joint displays (Guetterman et al., 2015) and interpretive comparison. We engaged in a qualitative coding of the coaching sessions to identify and classify the presence of BCTs using an established taxonomy (Michie et al., 2013). We conducted quantitative comparisons of the frequency and distribution of BCTs among dyads categorized by post-intervention levels of change in the Gingival Index (GI) Stable/Worsened, Improved, or Meaningfully Improved. This mixed-methods approach allowed us to examine how specific techniques varied by GI outcome group, providing both numerical trends and contextual insight into intervention delivery.

In accordance with the Good Reporting of A Mixed Methods Study (GRAMMS) recommendations (Emary et al., 2023), we specified the sampling, data sources, analytic procedures, and integration points across strands below. Quantitative data included repeated measures of gingival health outcomes, while qualitative data comprised transcripts of coaching sessions capturing real-time delivery of BCTs. Quantitative analyses focused on examining changes in gingival health across participants and grouping dyads by outcome patterns, while qualitative analyses involved systematic coding of transcripts to identify and categorize BCTs and examine patterns of use across sessions and dyads. The qualitative and quantitative strands addressed the same overarching research aim, were analyzed in parallel, and were integrated at the interpretation stage using joint displays to compare BCT patterns across outcome groups and to generate meta-inferences regarding their relationship to changes in gingival health.

### Participants

We included a subset of participants from the parent study in this mixed-methods analysis, specifically persons living with MD and their care partners who were randomized to Treatment Group 2 (Wang et al., 2025), which included the coaching intervention. A total of 20 dyads were randomized to Treatment Group 2, and 17 dyads completed the four coaching sessions. Thus, the current analysis focused on the 17 dyads from this group who completed all the coaching sessions. Participants and their care partner were included if the person living with MD was (a) age 60 or above; (b) had a diagnosis of MD within the past year; (c) had at least 4 teeth; (d) lived with an informal, unpaid, care partner who was willing to participate; (e) were community dwelling; and (f) were physically able to brush their own teeth. Participants were excluded if the person living with MD was unable to have an oral health evaluation due to illnesses or medications (Wang et al., 2025).

### Coaching intervention overview

The coaching intervention program was developed to enhance oral hygiene behaviors in persons living with MD, with support from their informal care partners. Drawing on the Adaptive Leadership Framework for Chronic Illness (Anderson et al., 2015), the intervention aimed to empower care partners to serve as effective facilitators in encouraging and assisting persons living with MD with cognitive decline in maintaining daily oral hygiene routines (Wu et al., 2021). The intervention received by Treatment Group 2 consisted of three core components: 1) a personalized oral care plan and training tailored to each dyad’s oral hygiene needs, 2) an educational booklet designed to improve knowledge of oral hygiene and dementia-specific strategies, and 3) a series of structured coaching sessions targeted at care partners to enhance their caregiving strategies, use of BCTs and leadership in promoting oral health by the persons living with MD. The coaching sessions concluded with the persons living with MD joining in the last 10 min of the coaching session to collaboratively set and review their oral hygiene goals. The overall intervention goal was to support and improve oral hygiene behaviors and outcomes for persons living with MD through guided involvement of their care partners.

The coaching modules (CM) component included four sessions: an initial home visit at baseline (CM1), followed by two telephone-based sessions during Week 4 (CM2) and Week 8 (CM3), and a home visit at Week 12 (CM4). Each session lasted approximately 30–45 min. These sessions were facilitated by an interventionist trained in BCTs and with prior experience in dementia care and caregiver coaching. During these sessions, care partners worked collaboratively with the interventionist to set and review SMART (Specific, Measurable, Achievable, Relevant, and Time-bound) goals related to the oral care for the persons living with MD. BCTs that guided the use of communication and cueing strategies (verbal, visual, and touch) to encourage and support persons living with MD in engaging in daily oral hygiene practices. During the home visits, a dental hygienist demonstrated proper oral care techniques and joined the coaching session to help co-develop an actionable oral health plan with the dyad and interventionist. These efforts emphasized both behavioral skill-building and partnership between the care partner and the person living with MD (Wu et al., 2022).

### BCT intervention implementation and fidelity

All coaching sessions were semi-structured and guided by an intervention manual, which was revised based on findings from our pilot study (Bryant et al., 2024). The manual explicitly outlined when and where to introduce specific BCTs during each session. A list of 17 prescribed BCTs was present in the intervention manual (See details in Table 1). While the manual provided a scripted structure to ensure fidelity, interventionists were encouraged to allow natural conversation flow and retain flexibility to introduce additional BCTs based on dyadic needs. This adaptive delivery was guided by the 93-item BCT Taxonomy v1 (Michie et al., 2013), enabling personalized engagement while maintaining protocol adherence. Protocol fidelity was monitored through routine audits of recorded sessions and ongoing training of interventionists across two sites. A senior investigator (RAA) conducted periodic reviews of randomly selected recordings to assess adherence to the manual and ensure consistent and accurate implementation of BCTs. In addition, interventionists participated in annual refresher training sessions to reinforce protocol adherence and calibration in BCT delivery.

**Table 1:** Behavioral change technique (BCT) codes and frequency across groups.

BCT codes (applied)	Overall (17 dyads) (%)	Stable or Worsened (≤0 no change or increase in GI) (5 dyads) (%)	Improved (0–25% reduction) (6 dyads) (%)	Meaningfully Improved (>25% reduction) (6 dyads) (%)
	
	*N *= 2,707	*n *= 1,066	*n *= 994	*n *= 647
**1. Review behavior goals^a^**	399 (14.7)	169 (15.9)	130 (13.1)	100 (15.5)
**2. Problem-solving^a^**	338 (12.5)	100 (9.4)	149 (15)	89 (13.8)
**3. Prompts cues^a^**	298 (11)	139 (13)	100 (10.1)	59 (9.1)
**4. Instruction on how to perform a behavior^a^**	252 (9.3)	91 (8.5)	97 (9.8)	64 (9.9)
**5. Feedback on behavior^a^**	225 (8.3)	118 (11.1)	68 (6.8)	39 (6)
**6. Goal setting behavior^a^**	203 (7.5)	90 (8.4)	50 (5)	63 (9.7)
**7. Action planning**	166 (6.1)	61 (5.7)	67 (6.7)	38 (5.9)
**8. Discrepancy between current behavior and goal^a^**	128 (4.7)	52 (4.9)	64 (6.4)	12 (1.9)
**9. Commitment^a^**	100 (3.7)	38 (3.6)	42 (4.2)	20 (3.1)
**10. Social support unspecified**	83 (3.1)	29 (2.7)	35 (3.5)	19 (2.9)
**11. Demonstration of the behavior^a^**	75 (2.8)	29 (2.7)	29 (2.9)	17 (2.6)
**12. Credible source^a^**	55 (2)	27 (2.5)	9 (0.9)	19 (2.9)
**13. Self-monitoring of behavior^a^**	48 (1.8)	14 (1.3)	12 (1.2)	22 (3.4)
**14. Behavioral practice rehearsal^a^**	45 (1.7)	17 (1.6)	16 (1.6)	12 (1.9)
**15. Social support emotional**	39 (1.4)	16 (1.5)	20 (2)	3 (0.5)
**16. Habit formation**	39 (1.4)	10 (0.9)	21 (2.1)	8 (1.2)
**17. Social support practical**	35 (1.3)	9 (0.8)	16 (1.6)	10 (1.5)
**18. Focus on Past Experiences^a^**	32 (1.2)	1 (0.1)	24 (2.4)	7 (1.1)
**19. Monitoring of behavior by others without feedback**	30 (1.1)	11 (1)	3 (0.3)	16 (2.5)
**20. Feedback on outcomes of behavior**	25 (0.9)	8 (0.8)	8 (0.8)	9 (1.4)
**21. Information about antecedents^a^**	20 (0.7)	5 (0.5)	11 (1.1)	4 (0.6)
**22. Information about health consequences^a^**	16 (0.6)	9 (0.8)	4 (0.4)	3 (0.5)
**23. Review outcome goals**	13 (0.5)	5 (0.5)	4 (0.4)	4 (0.6)
**24. Behavioral contract^a^**	10 (0.4)	7 (0.7)	0 (0)	3 (0.5)
**25. Information about social and environmental consequences^a^**	10 (0.4)	3 (0.3)	6 (0.6)	1 (0.2)
**26. Biofeedback**	8 (0.3)	5 (0.5)	1 (0.1)	2 (0.3)
**27. Monitoring outcomes of behavior by others without feedback**	7 (0.3)	0 (0)	6 (0.6)	1 (0.2)
**28. Self-monitoring of outcomes of behavior**	6 (0.2)	3 (0.3)	0 (0)	3 (0.5)
**29. Salience of consequences**	2 (0.1)	0 (0)	2 (0.2)	0 (0)

*Note*. GI = Gingival Index. Values report *n* (%).

a BCT codes present in the original intervention plan.

### Data source

#### Qualitative data

The qualitative data were drawn from 68 transcripts of intervention coaching sessions conducted with 17 dyads enrolled in Treatment Group 2 of the parent oral health intervention study (Wang et al., 2025; Wu et al., 2021). All coaching sessions were audio-recorded and professionally transcribed verbatim. Transcripts were imported into Dedoose, a secure, web-based platform that facilitated collaborative coding and team-based review. All transcripts were coded in the software to ensure data integrity and traceability throughout the analytic process.

Because this was a secondary analysis of a bounded dataset, sample size was determined by the number of dyads in the intervention arm who completed all four coaching sessions, rather than by iterative recruitment. Accordingly, we frame sample adequacy in terms of analytic (or informational) sufficiency rather than prospective saturation. The dataset included 68 longitudinal transcripts, providing repeated within-dyad observations over time as well as sufficient cross-dyad variation to examine recurring BCT patterns, role-based differences, and GI-related trajectories. Consistent with the concept of information power, where sample adequacy depends on the richness and relevance of the data to the study aim (Malterud et al., 2016), the longitudinal and interactional depth of the transcripts supported robust analysis. Themes were considered sufficiently developed when additional within-team comparisons no longer meaningfully altered the interpretive structure of the findings, consistent with approaches emphasizing analytic sufficiency over saturation in qualitative analysis (Braun & Clarke, 2021; Hennink et al., 2017).

#### Quantitative data

The primary quantitative outcome for this mixed-methods analysis was the Gingival Index (GI) (Löe, 1967), which was measured by a dental hygienist during clinical assessments at baseline, 3 months (following active phase), and 6 months (sustained change) (Wu et al., 2022). For the present secondary analysis, we focused on the change from baseline to 3 months, which corresponded to the end of the active 12-week coaching phase. The 6-month assessment in the parent trial was designed to evaluate maintenance of change and was not the primary endpoint for the current analysis. GI index scoring was calibrated annually between the two dental hygienists. In this analysis, we focused on the change in GI at 3 months, marking the end of the active intervention phase. GI was selected as the primary outcome as it is a validated and sensitive indicator of gingival inflammation that responds to plaque accumulation and, importantly, to changes in oral hygiene behaviors such as toothbrushing (Löe, 1967). Given that the intervention targeted daily oral hygiene routines and caregiving support for those routines, GI provides a clinically meaningful and behaviorally responsive outcome. Prior studies have demonstrated that gingival inflammation can show measurable improvement within 4–12 weeks following enhanced oral hygiene practices and behaviorally focused interventions, supporting the use of a 3-month follow-up period to detect meaningful change (Axelsson & Lindhe, 1981; Holloway et al., 2021). We categorized the participants into three groups based on the GI scores of the persons living with MD: Stable/Worsened (≤0 no change or increase in GI), Improved (0–25% reduction), and Meaningfully Improved (>25% reduction). While we sought to ground this classification in existing literature, we found no published guidance on what constitutes a clinically meaningful change in GI scores for this population. Thus, the threshold for Meaningfully Improved was established through team consensus based on clinical judgment of two team dentists and the distribution of the observed data. Qualitative analyses using an *a priori* BCT coding scheme allowed for an integrated mixed analysis in which we compared the types and frequencies of BCT use across the three GI outcome groups (See Table 1). In addition to GI scores, the frequency of BCTs coded within each coaching session served as a secondary quantitative indicator. Each transcript was reviewed using the BCT Taxonomy v1, and the number of each discrete BCT used during a session was recorded. This allowed us to quantify the intensity and variation in type of BCTs implemented across dyads and to explore patterns of BCT usage in relation to the GI outcome groups. This frequency-based coding enabled statistical comparisons of BCT delivery across clinical outcome strata, complementing our thematic analysis. Functional abilities were assessed using the Alzheimer’s Disease Cooperative Study–Activities of Daily Living (ADCS-ADL; Potashman et al., 2023). In this sample, ADCS-ADL scores were consistent with mild functional impairment, suggesting that participants generally retained sufficient capacity to engage in conversation and participate meaningfully in the oral health intervention.

### Data analysis

This study employed a secondary convergent mixed-methods analysis to examine how BCTs were implemented during oral health coaching sessions and how their usage related to participant oral health outcomes. The analyses of the qualitative and quantitative strands were conducted in parallel (Emary et al., 2023), then integrated at the interpretation and joint display stage (Guetterman et al., 2015) to explore how specific BCT patterns may be associated with changes in gingival health.

#### Qualitative analysis: BCT coding

We conducted a directed content analysis guided by Hsieh and Shannon (2005), using selected BCTs from the BCT Taxonomy v1 (Michie et al., 2013) as the predefined coding framework (See Table 1 for details). The analytic goal was to identify and classify discrete BCTs delivered by the interventionist or adopted by care partners or persons living with MD during each coaching session. A total of 10 team members participated in the coding process. All coders underwent structured training led by two BCT experts (RAA and DB), including independent practice coding of sample transcripts, iterative feedback, and weekly calibration sessions. Three coders (RAA, DB, CC) had completed formal BCT Taxonomy v1 certification. Training and calibration activities continued throughout the coding period to support consistency in code application. During formal coding, each transcript was independently double-coded by two coders (a primary and a secondary coder). Discrepancies were identified and discussed during weekly consensus meetings, with adjudication by a senior coder (RAA or DB) when consensus could not be reached. We did not calculate a formal inter-rater reliability coefficient; instead, we used a consensus-based coding approach supported by intensive training, ongoing calibration, double-coding, and audit trail procedures to enhance analytic rigor and consistency(O’Connor & Joffe, 2020). To minimize potential bias, coders were not informed of participant outcome group classifications during the coding process. Outcome-based comparisons were conducted only after coding was completed. To further ensure rigor and trustworthiness, we maintained an audit trail, conducted periodic independent audits, and engaged in team-based analytic review throughout the coding process (Guba, 1981).

#### Quantitative analysis: BCT frequencies and clinical outcomes

Once coding was complete, data were exported from Dedoose to R (version 4.4.3) for quantitative analysis. The primary clinical outcome was the GI, a validated indicator of gum inflammation, measuring change from baseline to 3 months (end of active intervention phase). We then quantified the frequency of BCTs used across all transcripts, calculating how often each BCT code appeared. Frequencies were summarized overall and stratified by (1) outcome group (GI trajectory) and (2) who the BCT was directed toward, i.e., person living with MD or care partner (See Table 1). This allowed us to explore how BCT application varied across dyads with different clinical responses and within different caregiver–care recipient dynamics.

#### Mixed-methods integration

Integration occurred at the analysis and interpretation levels through the construction of joint displays (Guetterman et al., 2015). These displays visually juxtaposed quantitative BCT frequency patterns with qualitative narrative excerpts to contextualize how and why certain techniques were more prevalent in dyads with meaningful oral health improvements (See Table 3). For example, frequently used techniques such as “Prompting Self-Monitoring” or “Demonstration of the Behavior” were linked with illustrative quotes showing how interventionists tailored delivery to individual dyads.

**Table 2:** Sample characteristics (*N *= 17 dyads).

Characteristics	Overall (17 dyads) (%)	Stable or Worsened (≤0 no change or increase in GI) (5 dyads) (%)	Improved (0–25% reduction) (6 dyads) (%)	Meaningfully Improved (>25% reduction) (6 dyads) (%)
**Dyad site**				
Duke University	13 (76)	2 (40)	5 (83)	6 (100)
New York University	4 (24)	3 (60)	1 (17)	0 (0)
**Participant age, Mean (*SD*)**	74.12 (6.82)	76.40 (8.53)	76.17 (6.34)	70.17 (4.62)
**Care Partner age, Mean (*SD*)**	71.53 (10.76)	64.20 (14.41)	77.33 (9.03)	71.83 (5.34)
**Participant sex**				
Male	9 (53)	3 (60)	3 (50)	3 (50)
Female	8 (47)	2 (40)	3 (50)	3 (50)
**Care partner sex**				
Male	6 (35)	0 (0)	3 (50)	3 (50)
Female	11 (65)	5 (100)	3 (50)	3 (50)
**Participant education**				
High school diploma or GED	2 (12)	1 (20)	0 (0)	1 (17)
Some college	6 (35)	3 (60)	1 (17)	2 (33)
College degree	5 (29)	0 (0)	4 (67)	1 (17)
Graduate degree	4 (24)	1 (20)	1 (17)	2 (33)
**Care partner education**				
Less than high school	0 (0)	0 (0)	0 (0)	0 (0)
High school diploma or GED	0 (0)	0 (0)	0 (0)	0 (0)
Some college	7 (41)	2 (40)	1 (17)	4 (67)
College degree	4 (24)	1 (20)	3 (50)	0 (0)
Graduate degree	6 (35)	2 (40)	2 (33)	2 (33)
**Participant race/ethnicity**				
Hispanic	1 (5.9)	0 (0)	1 (17)	0 (0)
Non-Hispanic Black	4 (24)	4 (80)	0 (0)	0 (0)
Non-Hispanic White	12 (71)	1 (20)	5 (83)	6 (100)
**Care partner race/ethnicity**				
Hispanic	0 (0)	0 (0)	0 (0)	0 (0)
Non-Hispanic Black	3 (18)	3 (60)	0 (0)	0 (0)
Non-Hispanic White	14 (82)	2 (40)	6 (100)	6 (100)
**ADCS-ADL score, Mean (*SD*)^a^**	62.00 (13.72)	60.20 (8.07)	61.50 (5.68)	64.00 (22.58)

*Note.* GED = General Educational Development; *SD* = standard deviation. Values report *n* (%) or mean (*SD*).

a ADCS-ADL = Alzheimer’s Disease Cooperative Study–Activities of Daily Living scale, a caregiver-reported measure of functional ability in basic and instrumental activities of daily living. Higher scores indicate less functional impairment (greater independence).

**Table 3:** Combined outcome for top behavior change techniques (BCTs).

BCT	Stable/worsened (%)	Improved (%)	Meaningfully improved (%)	Pattern
**Review behavior goals**	15.9	13.1	15.5	Consistently high across outcomes and roles
**Problem-solving**	9.4	15	13.8	Stronger use by those in improved outcomes and by care partners
**Instruction on how to perform a behavior**	8.5	9.8	9.9	Most evident in Improved/Meaningfully Improved groups; persons living with mild dementia received more instruction than care partners
**Prompts/cues**	13	10.1	9.1	More use in Improved groups and by care partners
**Goal setting**	8.4	5	9.7	More emphasized by persons living with mild dementia in the Meaningfully Improved group
**Action planning**	5.7	6.7	5.9	Frequently applied despite not being specified in the intervention manual; emphasized by care partners

This integrative approach allowed us to move beyond isolated findings by examining how the structure and intensity of BCT delivery may relate to improvements in clinical outcomes, while preserving the context of interpersonal dynamics between persons living with MD and their care partners. Through this, the study yielded actionable insights into how specific behavior change strategies function in real-world caregiving scenarios for persons living with dementia.

## Results

### Outcome overview

This mixed-methods analysis examined how BCTs were applied to compare differences in BCT use between oral health outcome scores (GI) and between use by the person living with MD and their care partner. Table 2 presents sample characteristics (See Table 2). Overall, we observed three key patterns: (1) A set of core BCTs was consistently used across all outcome groups, reflecting intervention fidelity. (2) Certain BCTs, particularly problem-solving and prompts/cues, were more prominent in dyads with improved outcomes. (3) BCT use differed by role, with care partners more actively engaged in implementation and adaptation, and persons living with MD more engaged in learning and goal-related processes.

As shown in Table 3, we conducted quantitative comparisons of the frequency and distribution of BCTs among persons living with MD on the GI index. Several BCTs, such as reviewing behavior goals, were consistently present across all outcome groups. Other BCTs, including problem-solving and prompts/cues, appeared more frequently in dyads with Improved or Meaningfully Improved outcomes. To contextualize these patterns, we applied qualitative coding to identify how BCTs were operationalized within the coaching sessions. We also explored how these techniques were distributed between the roles of persons living with MD and care partners. As presented in Table 4, some BCTs, such as reviewing behavior goals, were emphasized equally by both the person living with MD and the care partner, serving as a mutual anchor throughout the coaching process. Other BCTs showed clear role-based distinctions: care partners more frequently led problem-solving and provided prompts, while persons living with MD were more engaged in goal setting and learning how to perform a behavior. These role-based patterns help clarify how care partners and persons living with MD co-constructed the intervention experience.

**Table 4:** Role-based emphasis of behavior change techniques (BCTs) across dyads.

BCT code	Persons living with mild dementia (%)	Care-partner (%)	Role interpretation
**Review behavior goals**	14.3	14.9	Shared emphasis: review served as a mutual anchor
**Problem-solving**	8.7	13.8	Care partners led solution generation
**Prompts/cues**	5.0	13.1	Care partners provided external prompts and reminders
**Instruction on how to perform a behavior**	10.8	8.8	Persons living with mild dementia focused on the learning process
**Goal setting**	12.3	5.8	Persons living with mild dementia set goals; Care partners reinforced
**Demonstration**	1.4	3.3	Care partners modeled behavior more than persons living with mild dementia
**Action planning**	9.3	5.0	Care partners frequently supported planning although not part of the manualized BCTs

### Overall BCT use across all outcome groups

Across all dyads, several BCTs were used consistently, including review of behavior goals, problem-solving, instruction on how to perform a behavior, and prompts/cues, reflecting strong fidelity to the coaching protocol. For example, in a session focused on *reviewing behavior goal*s, care partners reminded persons living with MD of previously set oral care targets and checked in on progress. For example, two care partners recalled:

I would remind him that we’d set a goal to be more consistent with his oral care, that last time he’d said he wanted to do it at least four times a week and then I’d ask, ‘How’s that been going?’ (373, Care Partner, Stable or Worsened, CM2)

We check in each week, did we do it as planned? If not, we talk about what got in the way. (135, Care Partner, Improved, CM3)

*Problem-solving* frequently occurred in response to behavioral barriers. One care partner reflected, “Sometimes she resists, so I have learned to try a different approach, like changing the timing or making it more relaxed” (163, Care Partner, Meaningfully Improved, CM4). Such moments highlight the iterative and collaborative nature of problem-solving in the intervention.

*Instruction on how to perform a behavior* was another core BCT, typically used to model or guide specific behaviors. For instance, a coach reminded a care partner:

Okay. we are not sure that [the participant living with MD] is able to use the proper toothbrushing technique, in that the toothbrush needs to be at that 45 degree angle to the gum, and then you sweep down in the direction that the tooth grows on the top, and on the bottom jaw, you sweep up in the direction that the teeth grows. So, do you think that you would be able to use touch as a cue, to guide him in the proper adaptation of the toothbrush to the teeth? (373, Care Partner, Stable or Worsened, CM2)

These moments emphasized clarity and repetition to reinforce learning.

*Prompts and environmental cues* (BCT 7.1: Prompts/Cues) were also employed to initiate or reinforce behaviors. One care partner noted, “And getting into that routine, and I think those visual cues—like putting the toothbrush where she can see it—really helped her remember on her own” (373, Care Partner, Stable or Worsened, CM2). Another shared, “I use verbal cues like ‘toothbrush up, toothbrush down’ and demonstrate with my hand” (170, Care Partner, Improved, CM4).

*Goal setting* was commonly observed in early sessions, often in the form of negotiated, realistic targets. One care partner stated, “So we talked about starting with three times a week, right? That feels doable for now. We can build up if it goes well” (373, Care Partner, Stable or Worsened, CM2). Although not specified in the manual, *action planning* emerged frequently, particularly in response to failed attempts at behavior change. For instance, the dyad and coach collaboratively discussed when and how to implement the target behavior: “So, what would you like to start with for the next couple weeks?”; “Maybe mostly we would have to do it once a day in the evenings… We’ll work out a schedule… see how everything comes out.”; and “What do you think about being able to try to brush your teeth together three times a week?” (373, Care Partner, Stable or Worsened, CM2).

### Outcome group-based pattern

Dyads categorized as Meaningfully Improved on the GI showed clear alignment with the planned BCTs across their sessions, as their discussions and behaviors reflected that the BCTs were used as intended. Their quotes often illustrated coordinated routines, autonomy of the persons living with MD, and sustained engagement. For example, one care partner described, “We have a routine now. I set it out, and she brushes while I do mine. It helps. She’s calmer when it’s predictable” (506, Care Partner, Meaningfully Improved, CM2). The BCT, *instruction on how to perform a behavior*, also became embedded (“She tries it herself first. If she struggles, I help. But she likes doing it on her own now” [506, Care Partner, Meaningfully Improved, CM2]).

Dyads that *improved* but did not reach a “meaningful” level of change in the GI often demonstrated flexible adaptation of BCTs in response to daily challenges. *Action planning* emerged prominently in this group, despite not being specified in the manual. One care partner shared, “Last time we tried every day, and it didn’t work. Three times a week with reminders on the whiteboard—that’s what we’re doing now” (457, Care Partner, Improved, CM3). Another noted, “We figured out three times a week is more doable. I put a reminder on the fridge” (457, Care Partner, Improved, CM3). These examples show how care partners used the *action planning* BCT to experiment with and refine BCT use to support their routines and capacities.

*Emotional support* BCT also emerged in this group: “I tell her, ‘Let’s do this together.’ That way, she feels less alone. I don’t want her to think it’s just a chore” (457, Care Partner, Improved, CM4). The co-construction of routines and adaptive flexibility may have supported incremental progress even in the absence of deep instructional uptake.

In contrast, dyads whose outcomes were *Stable or Worsened* typically relied on coach-led BCTs without deeper adaptation or internalization. As one care partner admitted, “I remind her, but sometimes she just says no. I don’t push it. We skip it some days” (373, Care Partner, Stable or Worsened, CM2). Another reflected, “We said four times a week, but I honestly forgot too. I need to set an alarm or something” (373, Care Partner, Stable or Worsened, CM2). These accounts suggest more superficial engagement with the behavior change process and a lack of structured follow-through, such as that provided by action planning.

### Role-based patterns of BCT use

Care partners most often described using prompts/cues, problem-solving, reviewing, and reinforcing behavior goals, and, notably, initiating action planning. The transcripts of the coaching sessions revealed the care partner’s strong role in facilitating behavioral change in the person with dementia. The care partners demonstrated the use of the BCT but also how and why they adapted the BCTs to daily realities. For instance, one care partner explained, “I set the toothbrush by the sink after breakfast, so she sees it. Then I remind her after coffee. That’s our rhythm now” (135, Care Partner, Improved, CM3). Others demonstrated adaptive problem-solving: “Sometimes she resists. I’ve learned not to argue. I try something else, like singing the song we use” (163, Care Partner, Meaningfully Improved, CM4).

Coaching session data revealed that action planning was often described in concrete terms: adjusting frequency, placing visual reminders, or integrating oral care into existing routines. As one care partner put it, “We made a plan together. She likes afternoons, so now that’s when we do it. It fits better” (464, Care Partner, Improved, CM3). These behaviors suggest a high level of initiative and strategy development, even when not prompted by the coach.

Unspecified BCTs, such as emotional and relational support, were also described by care partners and appeared to complement instructional BCTs. One noted, “She gets embarrassed. So I always say, ‘It’s okay, we’ll try again tomorrow.’ I want her to feel safe, not judged” (506, Care Partner, Meaningfully Improved, CM2). These moments reflect relational adjustments that went beyond the original protocol but were instrumental in promoting persistence and confidence.

Persons living with MD, in contrast, more frequently discussed learning processes, especially in relation to instruction and goal setting BCTs. One participant stated, “She showed me again—how to move [the toothbrush] around in circles. I think I get it better now” (303, Person Living with MD, Meaningfully Improved, CM2). Another participant reflected on autonomy: “I told her I wanted to do it myself today. I don’t like being helped too much” (240, Person Living with MD, Meaningfully Improved, CM2).

Similarly, action planning BCT emerged as an important BCT that was not specified in the original intervention manual but was frequently facilitated by interventionists when working with care partners and persons living with MD. Although action plans were developed collaboratively, transcripts showed that interventionists often took the lead in structuring goals and ensuring they were framed as SMART (specific, measurable, action-oriented, realistic, and time-bound). For example, in one session, an interventionist guided the dyad by stating: *“*So our goal is going to be… brushing two times per day using verbal and visual cues. That sounds like a SMART goal to me.” (163, Interventionist, Meaningfully Improved, CM1). This goal-setting process took place during the final segment of the coaching session, when the dental hygienist joined the conversation to collaboratively create SMART goals in alignment with the intervention protocol.

## Discussion

We delivered a multicomponent intervention to care partners and persons living with MD to improve oral health behaviors and outcomes of the persons living with MD (Wu et al., 2021). This study used a mixed-methods design to analyze the BCTs taught to care partners of persons living with MD in an oral health intervention who then used the BCTs with the persons living with MD to improve their oral hygiene behaviors. We found that the most frequently applied BCTs across all dyads were review of behavior goals, problem-solving, instruction on how to perform a behavior, and prompts/cues. These BCTs were among those designated for use in the intervention manual, reflecting strong fidelity to the intervention design and adherence to the study protocol. We examined BCT use across the three GI outcome groups, as well as by the roles of care partners and persons living with MD, and found variation in the frequency with which BCTs were applied and by whom. Qualitative analyses also generated thematic findings derived from in vivo pattern coding beyond the *a priori* BCT coding scheme. These themes reveal intervention engagement that fell on the spectrum from active to more passive, meaningful dyadic coordination, and emerging routine building among dyads.

These merged data showed that care partners used the BCT consistently with the planned intervention and demonstrated their flexibility in how they enacted the techniques. In brief, we found that the use of review of behavior goals was high across all GI outcome groups and both roles (persons living with MD and care partners), functioning as a shared anchor that reinforced continuity of effort. Problem-solving was most evident in Improved dyads, suggesting its role in helping families adapt strategies to overcome barriers. Instruction on how to perform a behavior was particularly linked to Improved and Meaningfully Improved outcome groups, reflecting its alignment with learning and autonomy of persons living with MD. Prompts and cues were more frequently delivered by care partners, particularly in earlier stages, while goal setting emerged most prominently among persons living with MD in the Meaningfully Improved group, underscoring the value of ownership of achievable targets for persons living with MD. Beyond the planned techniques, we also observed repeated use of BCTs not specified in the manual, such as action planning and emotional support. Care partners actively employed action planning as they adjusted strategies to daily routines, for example, by moving reminders to a fridge and scheduling brushing at preferred times. Emotional support also emerged in the form of reassurance, often complementing other BCTs such as instruction and prompts/cueing. These findings suggest that, although fidelity to manualized strategies was strong, intervention effectiveness in real-world dyadic contexts was enhanced by adaptive use of complementary techniques that allowed families to tailor the intervention to their cognitive, emotional, and environmental realities. Collectively, these patterns highlight not only which techniques appeared most influential, but also the importance of having a shared, theory-informed taxonomy to specify both planned BCTs and emergent adaptations in a way that can be compared and built upon across studies.

The value of using the BCT taxonomy in the intervention design and delivery cannot be understated. Integration of the BCTs offered a systematic and reliable means for specifying the components of the intervention; thus, allowing our team to identify those components that may be serving as active ingredients for Improved and Meaningfully Improved GI outcomes (Michie et al., 2013) and to whom they may most effectively be delivered in a dyadic intervention. Interestingly, the findings align with some of the more pragmatic and simplified approaches to behavior change informed by the BCT taxonomy that involve just a few of the 93 possible BCTs. Using a limited number of BCTs has been found useful in a practice setting where time and the theoretical foundation for the taxonomy may be limited (Asimakopoulou et al., 2023). One such approach, the GPS (Goal setting, Planning, and Self-Monitoring) approach, has gained traction in recent years for positively impacting oral health behaviors and reducing clinical indices of periodontal disease (Asimakopoulou et al., 2023), and the goal setting and planning components of this simplified framework mirror the frequently used BCTs in our intervention. The component of the GPS approach that does not mirror the findings from this study is Self-Monitoring, which may be explained in that our intervention aimed to change oral hygiene behaviors among persons living with MD for whom self-monitoring may be difficult. Instead, the BCT *feedback on behavior* was more commonly used, reflecting the dyadic nature of our intervention (care-partner and persons living with MD) and the critical role care-partners play in helping persons living with MD monitor their behavior change. As is commonly recognized in implementation science realms, how and by whom an intervention is delivered may have as great or even greater impact on the outcome than the content of the intervention itself (Kolehmainen & Francis, 2012). Indeed, substituting a BCT such as *feedback on behavior* delivered by a care-partner instead of self-monitoring may be one of those critical adaptations needed in dyadic oral health interventions involving persons living with MD, extending the current literature, which has primarily focused on BCT interventions delivered to the individuals whose behavior change is targeted (Michie et al., 2013).

The integration of mixed-methods findings added depth to the quantitative patterns, pointing to mechanisms that may explain variation in GI outcomes. Three qualitative themes, levels of engagement (active vs passive), dyadic coordination, and routine building, cut across groups and highlighted the relational and contextual dimensions of change. Dyads in the Meaningfully Improved group often demonstrated active engagement, co-constructed routines, and autonomy of persons living with MD in oral care. For example, synchronized brushing routines or negotiated goal setting supported accountability and predictability. Improved dyads demonstrated adaptive flexibility, using action planning and problem-solving to tailor strategies, even when fidelity to the manual was less direct. Stable or Worsened dyads, by contrast, tended to rely on coach-led strategies without deeper adaptation, leading to more superficial engagement and missed opportunities for sustained change.

These patterns map closely onto the Adaptive Leadership Framework for Chronic Illness, which distinguishes between technical challenges (solved through clear instruction and structured tools) and adaptive challenges, which require iterative problem-solving, negotiation of roles, and the co-construction of new routines. Planned BCTs such as instruction addressed technical challenges, while the emergent use of action planning and emotional support reflected adaptive work that enabled dyads to integrate strategies into real life. This interpretation aligns with our prior studies (Corazzini et al., 2015; Shieu et al., 2023) applying the Adaptive Leadership Framework to oral health interventions for individuals with mild cognitive impairment and MD, in which adaptive capacity (flexibility and shared problem-solving) distinguished persons living with MD who benefited most.

Together, these insights suggest that effective BCT use is not solely about the presence of planned strategies, but about the capacity of dyads to co-adapt, reinforce each other’s efforts, and embed oral hygiene practices into shared daily routines. This underscores the importance of viewing BCTs not as static techniques but as relational tools that must be enacted collaboratively and flexibly to promote long-term sustainability.

### Limitations

This study has several limitations. Although we robustly examined changes in BCT use and oral health outcomes at baseline and 12 weeks, interpretation of the findings is limited by the absence of established guidelines for categorizing GI change. The three outcome categories (*Stable/Worsened*, *Improved*, and *Meaningfully Improved*) were derived from team dentists’ recommendations and the sample’s data distribution rather than clinically validated thresholds. Consequently, the analyses were intended to be descriptive rather than inferential, and the clinical significance of these categories should be interpreted with caution. Second, the frequency counts used to assess how many times each BCT code was applied across transcripts are partly dependent on the speech patterns specific to individual dyads. For example, if a particular dyad was more talkative or tended to repeat phrases, the frequency counts would be higher, and we were unable to account for speech patterns in the analysis. Finally, the study sample was small, although the study design generated very rich data with four intervention sessions conducted per dyad, producing ample text data from which the team could ascertain granular information.

### Implications for future research

These limitations notwithstanding, our findings have important implications for the growing body of evidence of how well-designed interventions using BCTs may improve oral health in this priority population. This formative evaluation revealed important un-manualized BCTs which should be incorporated to update the intervention manual and more fully explore their roles as active ingredients. Given the potential influence of certain BCTs in improved oral health outcomes, a fully powered RCT is warranted to establish the effectiveness of the behavior change intervention.

## Conclusions

This study demonstrates the value of integrating the BCT taxonomy in a mixed-methods analysis to better understand how behavior changes unfold in dementia caregiving dyads. While planned BCTs formed a strong foundation for fidelity, the most meaningful improvements were linked to dyads who actively adapted and co-owned strategies, coordinated their efforts, and embedded oral hygiene into daily routines. BCTs not specified in the intervention manual, such as action planning and emotional support, underscored the importance of flexibility in real-world applications. These findings extend the BCT literature by illustrating how dyadic, relational, and contextual factors shape the effectiveness of intervention strategies for persons living with MD. Future research should continue to refine and adapt BCT-driven interventions to ensure they are both feasible for care partners and meaningful for persons living with MD, ultimately improving oral health and quality of life in this vulnerable population.

## Data Availability

This study was not preregistered. Because the data consist of information from a small group of participants and are governed by Institutional Review Board (IRB) approvals and data use agreements, the de-identified datasets and full qualitative materials are not publicly available. Sharing these data outside the research team could compromise participant confidentiality in this vulnerable population.
